# Supplementary material for: MEDLINE search retrieval issues: A longitudinal query analysis of five vendor platforms
Source: PLoS One. 2021 May 6;16(5):e0234221. doi: 10.1371/journal.pone.0234221 (PMC8101950; doi:10.1371/journal.pone.0234221)
Supplement: Attachment — Submitted filename: Response-to-Reviewers.docx [file pone.0234221.s002.docx]

**Rebuttal Letter**

**Reviewer #1**

Reviewer #1: This well-written paper presents results from a study comparing search results from the MEDLINE database in five retrieval systems. The study examined differences in the number of documents returned by each system for 29 cases. The study is motivated by the need to better understand discrepancies between the systems and changes in the results within each system. The study is novel in that it collected comparative data over a twelve-month period. The study was carefully designed to make results comparable between systems, with the focus on constructing Boolean queries for each case such that the syntax of each query was modified to conform to the coding and syntax of each system while duplicating the same criteria precisely. The authors also endeavored to construct a set of cases that allowed for a diversity of query functions and data structures between the cases. The authors have provided access to their extensive dataset. The paper contributes an overview of differences in the number of results returned and hypothesizes informally on some factors that may explain differences observed.

The goal of the study is reasonable, and the data collected are detailed and extensive, however the paper falls short in four important dimensions. First, the design of the study is not explained and justified sufficiently, making it hard for this reviewer to fully assess the validity of the design; this may be corrected by clearer explanation. Second, analysis of the voluminous data is insufficient in two major respects: (1) descriptive information, including between and within system differences, lacks statistics and (2) explanatory analysis of differences is ad hoc, with insufficient attention to supporting generalization on the results. These problems may be addressed by more detailed and systematic analysis of the data and by revision to the flow of the results section. Third, the novelty and importance of the findings and conclusions are unclear; this may be remedied by additional discussions of existing literature on the question of obtaining sufficient coverage for systematic reviews. Fourth, the limitations of the study are not addressed by the authors, a shortcoming that can be remedied by inclusion of discussion on this topic. My review covers these issues in turn, ending with some additional comments on specific passages.

THE DESIGN
The presentation of the design is confusing and would be strengthen by more detail. Readers would be helped by a simple diagram showing the five systems, the concept of what I call a “case” (see below), the 29 cases, and the monthly data collection. It would also be helpful to list and define the data you collected and the variable(s) you are analyzing from that data.

The term “sets” as an organizing device is confusing. The 29 sets are “cases”, where each case represents a specific set of subject term(s) and delimiters. Only when I reviewed the data in GitHub was I able to understand the structure of the cases, which is important to understanding the results. Readers need more information on the structure and motivation for the cases used in the paper. This may be provided by summarizing not only the search parameters (table 2) but the content values (e.g., subject terms, delimiter values in addition to date).

**Author’s comment: We agree with the terminology suggestion and have made the changes throughout the manuscript. We have also dropped Table 2 (per a suggestion by the second reviewer) and have instead replaced that with your suggestion of using diagrams to describe the method. We have included two diagrams (Fig. 1 and Fig. 2). The first diagram breaks down the overall method that we used and the second diagram highlights the content values we used for various search parameters.**

In reviewing the data on GitHub I found that 28/29 cases cover only two topics (subject terms): neoplasm and dementia. The other case covers one author name. Each of the two topics was further qualified by publication, author, date, title, or related subject terms: immune, risk, and human. Motivation for the selection of these topic areas and the 29 cases are unclear. Why were these specific topics chosen? Why only two topics? What are the rationales for the specific variations in the construction of each case? This information is critical to your readers’ ability to assess the validity of your observations and conclusions. The key concern is that your design involves your direct control of the data you produced as your sample. For this reason you must be very explicit about the rationale for differences in the query constructions used in each case. Further, a systematic discussion of the similarities and differences in the 29 cases is essential because there is a great deal of overlap among the cases.

**Author’s comment: We extended a paragraph in the Methods section to describe the rationale for our choice of the terms. Our goal was simply to compose queries that contained combinations of keywords, specific fields, MeSH terms with one or more tree numbers, and MeSH terms that were exploded. Unless directly compared in the manuscript, the cases, despite using the same terms, are not comparable to each other. We only seek to compare within cases, as specified in the text. We re-used MeSH terms because the terms satisfied other critieria, such as they retrieved a substantial amount of results. Also as stated in the manuscript, our questions are driving us to seek whether differences appear in search result counts among platforms for a combination of basic search queries. Even if we were to use multiple MeSH terms, or unique ones for each of our cases, it is not possible to be exhaustive or comprehensive of the MeSH vocabulary. We simply need to show that there are substantial differences for basic queries.**

The very limited number of topics (subject terms) should also be explained. It has been long established that variability in search system results is highly dependent on the topic of searches used to compare systems (see list of references below). Generally, a comparative study with few diverse topics will yield biased results that misrepresent the average performance of a system over a given document collection. This is a limitation of the present study, thus the paper would be strengthened on why your approach is not biased by the two topics you chose. This goes back to the need to motivate your design more clearly. Better explanations are needed for why you used these specific topics and specific overlapping cases to compare the systems. You may also wish to compare your approach to other approaches such as using cases (topics/queries) from published systematic reviews.

**Authors’ comment: In addition to our comment above, we are familiar with the TREC research that you suggested below that discusses this but did not cite it because we did not think it relevant to this study. Those TREC studies that show that results are dependent on topics are testing different search strategies (different semantic queries) against the same corpus or collection (that may or may not be indexed differently). This is not directly comparable to this study where we test the same semantic queries (on a per case basis) across what is supposed to be the same document collection even though the document collection is hosted on different platforms. Our research here is showing that, on the contrary, these MEDLINE platforms are not quite the same document collection, despite being called MEDLINE. Here we show that results are a function of issues with the bibliographic records used by these platforms (which may be changed when imported by the information service provides into their platforms), the search fields offered by the various platforms differ (e.g., publication date specifity), and how each information service provider is indexing their version of the document collection.**

Also, in explaining your rationale simplified language would help, and where esoteric concepts are involved (e.g., term trees, exploding terms) these must be carefully explained for unfamiliar readers. For unfamiliar readers, understanding *why* functionality matters is essential. The details presented in Table 2 are unhelpful because the effect of each parameter is not explained clearly to readers. This goes back to the need to justify your use of the parameters in the cases you constructed.

**Author’s comment: We agree and have added text to help explain these concepts in the Methods section.**

Finally, you state that you are not testing the performance of the systems (line 172-173: “the queries were not designed to test user relevance or user needs”) but “basic functionality”. With this rationale the concept of “basic functionality” must be well defined. It must be related directly to the specifics of your case design and to the measure(s) you are using for comparison. Without this, readers do not have enough information to assess the validity of your approach.

**Author’s comment: We agree here too and have added text to help explain what we mean and have removed the confusing “basic functionality” language.**

DATA ANALYSIS
You have elected to compare the number of items returned by each of your total 1,740 queries (29*12*5). No summary or descriptive statistics are provided on these counts. Results are presented in independent line graphs for each system, with data from only a subset of the cases presented. Your discussion of “macro” changes is difficult to follow (starts line 209). A simple statistical between-systems comparison of counts would be easier to follow. These could be averaged and/or presented in relation to the structure of your cases.

**Author’s comment: With regard to presenting statistics, we decided to re-use the modified z-score that we applied in our first study and have provided two plots to help readers visualize the differences among cases per platform (note, we are still not comparing cases, just showing that there is a lot of variation across different types of queries). The plots also show the statistics for each case.**

The graphical presentation of results is very unclear. First, the magnitude of differences over time, and between cases and systems is represented or discussed. Charts are drawn with inconsistent y-axes in figures 1 through 4, and no chart shows the zero value on this axis. Without discussion on the magnitude, the charts exaggerate the scale of changes and differences, although the trend-lines are clear. Trends and differences may be presented in statistical forms more easily communicated to readers. Data from all cases should be presented, or clear rationale for selected presentation must be given so your reader can better assess the validity of your analysis, results, and conclusions.

**Author’s comment: We disagree about having to zero out the y-axis on the charts. Doing so would straighten out the trend line and make it appear as if there were no differences between the search results when comparing the same semantic query across platforms, and that is what would ultimately be misleading. Edward Tufte has made this point, too, regarding time series plots (**[**https://www.edwardtufte.com/bboard/q-and-a-fetch-msg?msg_id=00003q**](https://www.edwardtufte.com/bboard/q-and-a-fetch-msg?msg_id=00003q)**). However, we agree that greater context is needed and we have added some additional discussion about the magnitude in order to resolve any confusion about this. We have also added more discussion about the data. Figures 3-6 all focus on the plots with queries that were limited by publication date. These plots show that despite records having publication dates ending in 2015, the literature continues to grow and does so differently depending on the platform. We have tried to better clarify that in the manuscript. We have removed the original Figure 5 since that plot seems confusing and have replaced it with a new plot 7 and 8. Both of these show the modified z-score statistics mentioned above.**

As importantly, your “micro-view” analysis would be greatly enhance by a more systematic explanation tied directly to the rationale(s) for case construction. As presented, these results read as ad hoc, with selected issues demonstrated by selection of specific cases. This reader is left with questions about whether the cases were constructed as demonstrations of specific issues known to exist in comparisons between MEDLINE sources (e.g., Duffy, de Kock, Misso, Noake, Ross, and Stirk, 2016). For this reason it is very important to provide a more systematic analysis of between and within-system differences. For example, within the cases presented there appear to be some similarities in trends which might be used to classify sources of discrepancies. Some trends appear to be simply growth in the indexed literature. Questions about similarity/difference in index growth could be analyzed by examining duplication of items in each list returned by each system. Other trends seem to be errors (incomplete index load) or error correction (adding back missing records). Again, detailed analysis comparing the index records returned could be used to evaluate the magnitude and scope of error-related trends, and how these compare between systems. After estimating the effects of these potentially large factors on differences between and within systems, more specific differences due to functionality might be explored. As currently presented the contribution and meaning of all these sources of variability are unclear.

**Author’s comment: As noted above, the cases presented graphically are primarily cases that focus on queries with publication dates. These plots break the notion that the bibliographic universe is stable years after items have been published. Also, we disagree with the ad hoc argument. We state that we were interested in developing basic queries with few parameters in order not to overcomplicate our cases and therefore make it difficult to trace the causes for any discrepancies. We also stated in the manuscript the specific query criteria we sought to use and that are the basis of our queries, and these included queries that “contained some part or combination of the following search fields:” keywords, specific fields, MeSH terms with one tree number, MeSH terms with more than one tree number, and MeSH terms that were exploded.**

**You write that “some trends appear to be simply growth in literature,” but we clearly have shown that this does not explain everything, such as cases with queries with publication dates ending in 2015. We have also shown that other issues are at play, such as issues with how platforms interpret the publication date field. For example, PubMed and Ovid provide more control over date searches (Create Date, Entrez Date, Publication Date, etc) but the other platforms do not, and this may influence different results. And Web of Science/MEDLINE seems to completely fail for some queries.**

**Regarding duplication – this is a difficult problem to answer especially with search results that return millions of records. We did not attempt to do that in this project and intentionally only looked at raw search result counts. We would like to examine the records more closely in a follow up project, but we do not believe that examining search result counts solely is an invalid method.**

Finally, you have elected to analyze only the quantity of items returned, and specifically to forgo analysis of results quality. The analysis would be greatly strengthened by an argument on why quantity is more important than quality, or at least, how quantity relates to quality.

**Author’s comment: We don’t think there’s an argument for why quantity is greater than or less than quality in results. This is an ongoing project and the first part of tackling what is largely an underresearched area. This not simply a test of database functionality but a test of whether MEDLINE is MEDLINE is MEDLINE (depending on the platform), and it is important to know that since MEDLINE, broadly speaking, is an important database for certain kinds of studies, such as systematic reviews. Our first goal in this project is thus to continue getting baseline measurements for the MEDLINE discrepancies across platforms. Also, most research that we’ve seen that is related, including some of the references that you suggest in your related findings list, are comparing apples to oranges, which is partially one reason we were motivated to do this study. For example, in some of the references to related findings that you suggested below, the authors of those papers are comparing PubMed to Ovid-MEDLINE, but PubMed is more than MEDLINE, as your link to the FactSheet shows (**[**http://wayback.archive-it.org/org-350/20180312141605/https://www.nlm.nih.gov/pubs/factsheets/dif_med_pub.html**](http://wayback.archive-it.org/org-350/20180312141605/https://www.nlm.nih.gov/pubs/factsheets/dif_med_pub.html)**). The Duffy et al (2016) article does show that it is good to search PubMed NOT MEDLINE, but aside from our COVID-19 example, this is not directly comparable to our study since we’re strictly comparing MEDLINE to MEDLINE across platforms. The Katchamart et al. (2011) article does not actually test sensitivity because they compare PubMed to Ovid-MEDLINE and not PubMed-MEDLINE to Ovid-MEDLINE, which is again problematic because PubMed is more than MEDLINE.**

MEANING OF RESULTS
Throughout the paper the question of “why” differences between systems matters is not completely clear. The paper would be greatly strengthened by discussion of the implications. Why do these findings matter? For whom do they matter? Are expert searchers aware of these differences? What do experts do to compensate? Evidence from the literature on how clinical decision-making, systematic review, knowledge bases are adversely impacted by differences would enhance readers’ understanding of the importance of these results. More specifically, how does each type of difference discussed relate to risks and adverse outcomes related to incomplete or erroneous information?
Importantly, some of the existing literature on differences between systems has not been cited. Please see the list below. These studies have found similar differences between and within systems. Relating your results to the prior findings would greatly strengthen the paper.

**Author’s comment: We agree that the manuscript would be strengthened by addressing these questions and have added text to do so.**

LIMITATIONS
The paper has no discussion on the limitations of the study. This is needed so that readers can understand how to apply the findings in a valid and trustworthy fashion.

**Author’s comment: This was an oversight on our part and we thank the reviewer for pointing out that we missed adding a discussion of the limitations. This has been added.**

Other comments:
Associate the example in table 1 with the information you provide in the text (lines 171- 181). Connect the example to the field names and explain how each relates to the concept of “functionality”
Drop table 2. The information is not useful to readers. More useful would be a more general explanation of why differences in these specific parameters matter and how they relate to the rationale for construction of your cases.

**Author’s comment: We agree. We have dropped Table 2 and have added, per your suggestion, a diagram of the method used here. We have also added some discussion about the functionality issue, and we thank you for pointing out these shortcomings. They are helpful.**

References on related findings:
Boeker M, Vach W, Motschall E. Semantically equivalent PubMed and Ovid-MEDLINE queries: different retrieval results because of database subset inclusion. J Clin Epidemiol. 2012 Aug;65(8):915–6. (See the paper discussed below). Note that the authors here describe a discrepancy created by an incorrect specification of an OVID query.)
Boeker M, Vach W, Motschall E. Time-dependent migration of citations through PubMed and OvidSP subsets: a study on a series of simultaneous PubMed and OvidSP searches. Stud Health Technol Inform. 2013;192:1196.
Duffy, S., de Kock, S., Misso, K., Noake, C., Ross, J., & Stirk, L. (2016). Supplementary searches of PubMed to improve currency of MEDLINE and MEDLINE In-Process searches via Ovid. Journal of the Medical Library Association: JMLA, 104(4), 309.
Duffy, S., Misso, K., de Kock, S., Noake, C., Ross, J., & Stirk, L. (2016, June). Supplementary searches of PubMed to improve currency of MEDLINE (Ovid) searches for systematic reviews: time taken for a record to move to MEDLINE. In 15th EAHIL Conference (pp. 6-11).
Kaunelis D, Farrah K, Severn M. Canadian Agency for Drugs and Technologies in Health. The missing 2%: PubMed NOT MEDLINE. Presented at: Canadian Health Libraries Association/Association des bibliotheques de la sante du Canada (CHLA/ABSC) 2010 Jun; ; Kingston, ON, Canada; 7–11.
Katchamart W, Faulkner A, Feldman B, Tomlinson G, Bombardier C. PubMed had a higher sensitivity than Ovid-MEDLINE in the search for systematic reviews. J Clin Epidemiol. 2011 Jul;64(7):805–7.
Lu, Z., Kim, W., & Wilbur, W. J. (2009). Evaluation of query expansion using MeSH in PubMed. Information retrieval, 12(1), 69-80.
US National Library of Medicine. Fact sheet: MEDLINE, PubMed, and PMC (PubMed Central): how are they different. [Internet] Bethesda, MD: The Library; 2016 [updated 6 Jan 2016; cited 2 Dec 2016]. < https://www.nlm.nih.gov/pubs/factsheets/dif_med_pub.html>.
Wanner, A., & Baumann, N. (2019). Design and implementation of a tool for conversion of search strategies between PubMed and Ovid MEDLINE. Research synthesis methods, 10(2), 154-160.

References on topic variability and system comparisons:
Banks, D., Over, P., & Zhang, N. F. (1999). Blind men and elephants: Six approaches to TREC data. Information Retrieval, 1(1-2), 7-34.
Robertson, S. E., & Kanoulas, E. (2012, August). On per-topic variance in IR evaluation. In Proceedings of the 35th international ACM SIGIR conference on Research and development in information retrieval (pp. 891-900).
Sakai, T. (2016). Topic set size design. Information Retrieval Journal, 19(3), 256-283.

**Reviewer #2**

Reviewer #2: I really enjoyed reading this manuscript. I appreciate the amount of work the researchers put into the project to collect data over a year with repeated searches in 5 bibliographic databases to track the search results. The analysis focused on identifying the inconsistent outputs and the causes of such issues across databases. The findings are important because missing publications can be life or death for a medical situation. The researchers also added the new topic CoViD-19 in the study, which is a plus.

I have only minor suggestions for consideration to help strengthen the manuscript:

In the Conclusion section, you wrote, "If this criticism is valid for the MEDLINE platforms, our results may strengthen the recommendation by Cochrane [23] that no single MEDLINE platform should be considered a sole source." Although you are hedging the findings and advice, I still hope to hear more than echoing Cochrane. Are there search strategies you would recommend for searchers to handle specific databases and cross database validation of search results? Further, what improvements the database providers should make? I think an important outcome of your results is to guide also practice in addition to understanding the phenomena.

**Author’s comment: We agree and have strengthened our discussion and final sections to help bring these issues home.**

I wondered if Figure 5 could have the white and black bars for the same search set side-by-side (i.e., the two bars next to each other) and also add data labels for easy visual comparison.

**Author’s comment: Based on the comments of the other reviewer, we decided to remove the original Figure 5 and replace it with more detailed figures based on the modified z-score. This is a method that we used in our first study.**

editing:

(The paper reads well-written. These perhaps more a stylist preferences by different readers.)

line 57, change "full text sources" --> full-text sources

**Author’s comment: Fixed**

Line 116, "This is commonly known problem" [add "a" before commonly]
**Author’s comment: Fixed**

Line 163, "on a per set basis" --> on a per-set
**Author’s comment: Fixed**

Line 182 - 185 "We added date ranges to test whether search result counts would remain frozen after the end of the publication date and to add an additional control that would help explain differences between the platforms." would read clearer if rephrased or illustrated with the actual queries.

**Author’s comment: We added text that refers to Table 1 as an example.**

Line 189, "field specific keywords" --> field-specific keywords

**Author’s comment: Fixed**

Line 190, the same need to hyphenate the two words field-specific

**Author’s comment: Fixed**

Line 217, "on a per query basis" --> on a per-query basis

**Author’s comment: Fixed**
