# Supplementary material for: MEDLINE search retrieval issues: A longitudinal query analysis of five vendor platforms
Source: PLoS One. 2021 May 6;16(5):e0234221. doi: 10.1371/journal.pone.0234221 (PMC8101950; doi:10.1371/journal.pone.0234221)
Supplement: Attachment — Submitted filename: Response-to-Reviewers.docx [file pone.0234221.s003.docx]

PONE-D-20-15022R1
Methodological Issues with Search in MEDLINE: A Longitudinal Query Analysis
PLOS ONE

Dear Dr. Burns,

Thank you for submitting your manuscript to PLOS ONE. After careful consideration, we feel that it has merit but does not fully meet PLOS ONE’s publication criteria as it currently stands. Therefore, we invite you to submit a revised version of the manuscript that addresses the points raised during the review process.

As you can see in the review report at the end of this email. Both reviewers agree that it is a significantly improved version; however, they have raised some issues regarding the presentation of results, literature, and what could be the recommendations/application of these findings for MEDLINE or other similar vendors/databases. These comments would be helpful while revising the manuscript.

Please submit your revised manuscript by Nov 26 2020 11:59PM. If you will need more time than this to complete your revisions, please reply to this message or contact the journal office at plosone@plos.org. When you're ready to submit your revision, log on to https://www.editorialmanager.com/pone/ and select the 'Submissions Needing Revision' folder to locate your manuscript file.

Please include the following items when submitting your revised manuscript:This is an important and major assignment. Please submit soon. The second Wikipedia assignment builds off this one.

- A rebuttal letter that responds to each point raised by the academic editor and reviewer(s). You should upload this letter as a separate file labeled 'Response to Reviewers'.
- A marked-up copy of your manuscript that highlights changes made to the original version. You should upload this as a separate file labeled 'Revised Manuscript with Track Changes'.
- An unmarked version of your revised paper without tracked changes. You should upload this as a separate file labeled 'Manuscript'.

If you would like to make changes to your financial disclosure, please include your updated statement in your cover letter. Guidelines for resubmitting your figure files are available below the reviewer comments at the end of this letter.

If applicable, we recommend that you deposit your laboratory protocols in protocols.io to enhance the reproducibility of your results. Protocols.io assigns your protocol its own identifier (DOI) so that it This is an important and major assignment. Please submit soon. The second Wikipedia assignment builds off this one.can be cited independently in the future. For instructions see: [http://journals.plos.org/plosone/s/submission-guidelines#loc-laboratory-protocols](https://nam04.safelinks.protection.outlook.com/?url=http%3A%2F%2Fjournals.plos.org%2Fplosone%2Fs%2Fsubmission-guidelines%23loc-laboratory-protocols&data=02|01|sean.burns@uky.edu|a80f1f9dac874337351f08d86e97296c|2b30530b69b64457b818481cb53d42ae|0|0|637380942806022403&sdata=URiftD2FV4eYSsctBM7V9Wff6KmIrCfGkBT7gAqQKPg%3D&reserved=0)

We look forward to receiving your revised manuscript.

Kind regards,

Muhammad A. Z. Mughal, PhD
Academic Editor
PLOS ONE


[Note: HTML markup is below. Please do not edit.]

Reviewers' comments:

Reviewer's Responses to Questions

**Comments to the Author**

1. If the authors have adequately addressed your comments raised in a previous round of review and you feel that this manuscript is now acceptable for publication, you may indicate that here to bypass the “Comments to the Author” section, enter your conflict of interest statement in the “Confidential to Editor” section, and submit your "Accept" recommendation.

Reviewer #1: (No Response)

Reviewer #3: (No Response)

2. Is the manuscript technically sound, and do the data support the conclusions?

The manuscript must describe a technically sound piece of scientific research with data that supports the conclusions. Experiments must have been conducted rigorously, with appropriate controls, replication, and sample sizes. The conclusions must be drawn appropriately based on the data presented.

Reviewer #1: Yes

Reviewer #3: Partly

3. Has the statistical analysis been performed appropriately and rigorously?

Reviewer #1: Yes

Reviewer #3: I Don't Know

4. Have the authors made all data underlying the findings in their manuscript fully available?

The [PLOS Data policy](https://nam04.safelinks.protection.outlook.com/?url=http%3A%2F%2Fwww.plosone.org%2Fstatic%2Fpolicies.action%23sharing&data=02|01|sean.burns@uky.edu|a80f1f9dac874337351f08d86e97296c|2b30530b69b64457b818481cb53d42ae|0|0|637380942806032400&sdata=V8x78onWhVwxGM9MXnR8JWpsT6FZhkLbNiffGaWxrX0%3D&reserved=0) requires authors to make all data underlying the findings described in their manuscript fully available without restriction, with rare exception (please refer to the Data Availability Statement in the manuscript PDF file). The data should be provided as part of the manuscript or its supporting information, or deposited to a public repository. For example, in addition to summary statistics, the data points behind means, medians and variance measures should be available. If there are restrictions on publicly sharing data—e.g. participant privacy or use of data from a third party—those must be specified.

Reviewer #1: Yes

Reviewer #3: Yes

5. Is the manuscript presented in an intelligible fashion and written in standard English?

PLOS ONE does not copyedit accepted manuscripts, so the language in submitted articles must be clear, correct, and unambiguous. Any typographical or grammatical errors should be corrected at revision, so please note any specific errors here.

Reviewer #1: Yes

Reviewer #3: Yes

6. Review Comments to the Author

Please use the space provided to explain your answers to the questions above. You may also include additional comments for the author, including concerns about dual publication, research ethics, or publication ethics. (Please upload your review as an attachment if it exceeds 20,000 characters)

Reviewer #1: Second review
The authors have made substantial and helpful changes to the paper, which greatly improve readability and clarity. The materials and methods section is greatly improved with the additional explanation of the queries and the two diagrams. The new limitations and future research sections are excellent. Below I’ve mentioned some important changes to further enhance clarity and a section that needs an extensive revision (lines 229 through 274).

You’ve used the term “sets” to mean different things. Clarifying further with some minor changes would make it easier to follow. Where you are referring to results returned by a query call these “results sets” (or retrieval sets, but be consistent). Where you are referring to the queries comprising a case, call these “query sets”. There are a lot of places where “sets” is confusingly ambiguous. Being much more specific about what you are referencing with the term “sets” would make the paper easier to read and understand. I suggest that where possible you drop the term “set” and say specifically what you are talking about. See specific suggestions below.

**Thank you for this suggestion. We agree. Instead of globally replacing ‘sets’ with ‘query sets’ or ‘results sets’, though, we went through the manuscript and removed some instances of the term, replaced some with the term ‘cases’, where appropriate and based on the suggestion in a prior review, replaced the term with other wording, as appropriate, and sometimes simply removed the term and modified the sentence in other ways. We believe the manuscript is clearer now.**

The initial presentation of results is still very difficult to read and follow. This is an important part of the paper and it needs to be much clearer, particularly when you refer to comparisons such as “within”, “between” and “over”.

You are using Macro and Micro to organize the presentation, but these are not defined for the reader and the terms are not informative. Use your intro paragraph to explain what you will be presenting as results. As I understand it from the abstract, there are four main findings. WITHIN results from each individual platform, online-first publishing creates metadata differences that cause changes in results sets over time for a single work. Three causes are found for differences in results sets BETWEEN platforms: 1) differences in search field specificity causes differences between platforms; 2) differences in the timing of file updates causes differences between platforms; and 4) general data integrity problems cause differences between platforms. Presenting the results as being about either changes over time WITHIN a platform, or changes over time BETWEEN platforms, would make it much easier to understand.

**Thank you for this suggestion. We have revised the intro to clarify what we mean by macro and micro and why we are using these two perspectives to look at the data.**

Your first two sentences in the results section are extremely hard to understand: (line 238) “A macro examination of the search cases restricted by publication date (cases #03–#11) mainly indicate that there are only substantial differences in total search result counts over time between platforms within each set, for example, between WSML and PMML (Figure 3, top left plot). This macro view indicates that although there are differences among platforms with respect to absolute differences in search result counts, the trends are reliably comparable across time on a per-query, per-platform basis (Figure 3).” I’ve read through this several times and tried, but I do not understand your point.

**We have revised the intro to the Results section and have added a short explanation of the macro and micro perspectives and what they have to offer.**

The multitude of charts (which are so small they are nearly indecipherable) are not helping, nor is the rest of the paragraph. The heading for Figures 3 and 4 are not informative. The headings need to tell the reader what they are looking at. Because the legend for Figure 3 cannot be read (it is extremely blurry) the whole figure is not useful and it creates confusion, particularly when compared with Figure 4. Figure 4 seems to show the same data (counts by platform over time for separate cases), but in F3 the counts appear to be constant while in F4 the counts vary.

**This is the key point of these charts. They show the same data but at different views, and it is these different views which are one of the main findings of this paper. We have altered and revised the text to make the difference between the macro and micro views clearer and to highlight their role in this paper. Fig 3 shows that these platforms do not appear all that different when looking at them from up high, or macro, but Fig 4 shows that when the same data is examined up close, they don’t trend the same way, even though each panel should, theoretically, be exactly the same.**

**We are not sure why the charts are reading as indecipherable. They conform to the plot specifications required by PLOS ONE and we uploaded files with the resolution of 2250 x 1266 pixels. They are not blurry on our systems. All we can do is double check with the manuscript submission process that the files are rendered correctly. However, we agree that the legend for Fig 3 was too small and not readable enough, and we have fixed that.**

Readers need a clear explanation, in simple language, of what is depicted in both figures, why they are different, and why it matters. I still believe you do not need most of your figures, particularly those that are truly unreadable. The information in Figures 3, 4, and 5 could be best represented with a small set of exemplars for each type of trend.

**We think these plots are important and central to the main findings of this paper. However, we have revised the Figure captions and added more detail about what the plots convey, and this detail is influenced by your suggestion above about the micro and macro views. We have also replotted the data to include better legends.**

I suggest you take the reader through one full example of a case and explain each figure as you go. Generally, lines 229 through 274 should be revised extensively. As food for thought, your rebuttal comments are much clearer than the paper. There you have written in clear, more direct language that communicates your points more effectively.

**Thank you for this suggestion. We have heavily revised this part of the manuscript. We borrowed some of the language from our rebuttal and more completely introduced and framed the results.**

Examples of ambiguity in the use of the term “sets”
In the abstract: line 17 “We found that search results vary considerably depending on MEDLINE platform, both within sets and across time.” ”… within sets …” is very unclear. Do you mean “… within results sets….” or “within cases” or something else?

**We have revised this.**

Line 37 “queries influence information retrieval sets were once common lines of inquiry” Clarify that this as “retrieval results sets”.

**We fixed this.**

Line 127 “…. influence different search behaviors and retrieval sets [45,46].” Clarify that this as “retrieval results sets”.

**Fixed.**

Line 132 “… evaluating recall and precision on the retrieval sets in these systems”. Clarify that this as “retrieval results sets”
**Fixed.**

Line 172 “…equivalent to each other on a per set basis.” Clarify this as “equivalent to each other on each platform. Throughout the paper we refer to the equivalent queries as query sets (italics).”

**Fixed, but since we are using the word cases now, we have modified the above to reflect that.**
Line 180 Table 1 refers to “Search Set #” . Retitle the column as “query set #”. The whole table should be labeled something like “Example Case showing semantically equivalent queries for the five platforms”.
**Fixed.**

Line 202 “…in order to compare search count results based on logical sets.” This is unclear. As I read it, you wanted to “…compare each platform’s Boolean processing”
**Fixed.**

Line 214 “…a comparison of search result counts within each set”. This confusing. “Within sets” obscures the fact that you are comparing the platforms. You are comparing BETWEEN the results sets of each platform for each case.
**Fixed.**

Line 229 “Our query sets include 100 queries without publication date ranges and nine sets containing..” Clarify as “… and nine cases containing ….”
**Fixed.**

Line 234 “…the differences we found across the search sets,…” Clarify – are these differences found BETWEEN platforms or differences found WITHIN platforms?
**Fixed as part of the major revision to this section.**

Line 337 “This relative consistency across platforms was found in other search sets that included additional…” Clarify as “…was found in other cases that included…”
**Fixed.**

Line 355 “…although search result counts were different among these platforms within these sets,…” Clarify as “among these platforms for these cases….”
**Fixed.**

Line 363 “…initially within a reasonable range of the other platforms in the respective sets, but then..” Clarify by simplifying and saying “within a reasonable range of the other platforms, but then…”

**Fixed.**

Reviewer #3: My comments to the authors are as follows:

- As a biomedical librarian. I experiment regularly with the search functionality of many platforms.
- I also have performed complex search queries on the platforms used in your query analysis (PubMed, ProQuest, EBSCOhost, Web of Science, and Ovid) and have made cross comparisons over the years.
- I can tell you that these problems are not new; however, I concur anecdotally with what you have proven in your manuscript and your year long + longitudinal analysis. I did not spend any time checking the accuracy of the statistics.

**We have heard the same anecdotal stories and share similar experiences. The motivation for this study was to document these issues in order to have strong evidence for the problems and not just anecdotal support. Your comment below is noted about the importance of strengthening suggestions for searchers, but we also think the problem 1) lies with the vendors, their documentation, their implementation of MEDLINE, and more, 2) publishers who use multiple publication dates that only serve to complicate the bibliographic record simply to have articles assigned to volume and issue numbers someday down the line, and 3) the lack of attention paid to this problem and the need for more studies not just by us but by others in the field. We think the issues with these databases that we report here could raise serious, clinical problems for data based on items retrieved from these databases, including systematic reviews. We have added some text or strengthened some of our recommendations to help clarify this.**

- In using those platforms, I have also observed that controlled vocabulary searches across PubMed, EBSCOhost & Ovid are mostly similar / consistent in terms of retrieval numbers (especially back dating) and that those searches can be translated into the different syntax quite reliably. Searches involving current publication years and current annual indexing always produce varying numbers.

**Yes, we hope that this helps document inconsistencies and consistencies among MEDLINE platforms.**
- Further, any type of searching involving author supplied keyword fields, searching in the titles and abstract fields, or any type of adjacency and frequency searching introduces varying and confusing numbers in all platforms. As you know, PubMed for example does not permit proximity / frequency searching although there is a rumour going around on Twitter that it is possible in the new PubMed interface.

**Yes, again, we think this has been poorly documented when MEDLINE platforms are compared to each other.**
- Given your paper's findings and what it adds to the body of evidence in searching Medline, what is your recommendation to searchers? You don't seem to address this at all in your conclusion.
- How should this evidence inform our work?

**Honestly, we do not think we know enough yet to make a recommendation like this. In our conclusion, we recommend potential research projects that need to be undertaken. We seriously believe that a comparison among MEDLINE systems is understudied but that it reveals problems not just between systems but in each system. More complex queries, as you comment below, would be worthwhile, but this baseline documentation is important, we think, where we start to measure how these platforms behave with very simple queries. The more complex the query, as we note in the paper, the more difficult it will be to track problems in these systems.**

Should we simply expect different results as we move across platforms and remember to watch for these in performing simple searches? Is that the message? I can tell you with certainty that most searchers are aware of this type of deficiency and how the problems shift over time. In other words, it's not news and the problems you uncovered are not stable and will be different next year.

**Again, it’s not well documented. We also agree that many expert searchers are aware of these problems anecdotally. We also have the kinds of personal experiences with these platforms, which is what motiviated this study. But until this is scientifically documented, as we have tried to do here and in our JMLA paper, anecdotal evidence and personal experience are scientifically insufficient, and thus have little chance to support change in these systems, however that change might look once these issues are sorted out more. We do think that such change might involve some standardization and that this might be aided by standards organizations like NISO as well as the creators of these systems, like NLM, and other stakeholders, like librarians, medical professionals, and more.**
- Further to your findings, I'm unclear about generalizability to other search queries. You mention that, in creating your search queries, you've given attention to queries that are semantically similar overall. I'm not clear what you mean; are these search queries generalizable to other sets of queries, and as such do they reveal broader patterns for each of the platforms? Further, would you expect to find similar patterns and results if you tested a different set of 125 queries?
**We do not think that generalizability is important here in the standard inferential sense. What we think is important here is that our confidence in these systems is not well warranted if they behave as inconsistently as we have documented with these 29 cases of queries and after 12 months of data collection. We are also not sure if generalizability is possible here. Even if we detect patterns in these inconsistencies between platforms, database technology changes, scholarly publishing changes, and more. There is simply too much randomness to infer from specific patterns over time.**

- Can I extrapolate from your findings that there is, thank goodness, more consistency across Medline platforms when the search queries are complex (for example, during multi-set systematic review searching)? If so, that would be worth emphasizing or at least surmising yes or no in the conclusion for the simple reason that it will reassure systematic searchers.

**We are honestly not sure. We seriously think more research needs to be done. This is because one complex search is not like the other. For example, does complex mean that a query simply mixes search fields, includes one or more Boolean operator, includes a mix of controlled terms and string-based searches? We do not know. We think the importance of this paper is that it raises doubts about this and to, hopefully, provide some justification for more research. We added some text to help clarify this.**

I read a similar paper you authored published last year in the Journal of the Medical Library Association: Burns CS, Shapiro RM, II TN, Huber JT. Search results outliers among MEDLINE platforms. Journal of the Medical Library Association: JMLA. 2019 Jul;107(3):364.
- In comparing the two papers, apart from testing the searches over time, how much of the concept and analysis here are expanded upon and/or different from your 2019 paper? You do allude to similar work you have performed in the past early in your manuscript but you don't discuss this paper specifically unless I missed it. The titles of the two papers are different; are they, in your view, sufficiently different / distinct both in terms of conceptual design and methods to warrant two separate manuscripts? If they are connected, by virtue of concept or overall goals for your research, you should say so.
- Further, the JMLA paper in 2019 is well-written though the PLoS manuscript is more detailed and of course deals with the longitudinal aspects. The title of the 2019 manuscript describes the paper accurately; it would be worth adapting that title to your PLoS manuscript, using the same economy of words and approach, to connect the two papers. The current title does not in my view convey the subject content of the manuscript in a descriptive way.

**We agree and thank you for the suggestion and for reading the prior paper. We have added additional text in the methods and conclusion sections to build off that JMLA paper and have changed the title of this paper to better reflect the link between the two.**

- In comparing your two efforts, I'm aware of added sections in the current manuscript pertaining to calendar year (2020) with regard to searches for COVID-19. That is timely and potentially useful but the question is still whether this adds substantively to our understanding.

**It shows that the MEDLINE platforms are not updated equally and at different rates. Again, we think that if this is known before, it’s only known anecdotally, and it’s important to document this scientifically. We think that’s very important for understanding big differences between these platforms and why it might be especially important to cite the specific MEDLINE platform used in studies that depend upon them for data. We have made that statement clearer in our conclusion.**

On line 396, you indicate that Medline "contains more than 25 million references to journal articles" but this number is by now in 2020 considerably higher than that, and possibly now as high as 28 million.
- For this review, I have had the privilege of being able to read the previous peer reviewers' comments of your paper, and your responses.
- I agree with the previous peer reviewers' comments generally but in particular want to emphatically agree with the comments / question pertaining to how you devised the search queries. It's unclear to me, even after reading the methods section again, why you chose to focus on the queries you did as a basis for testing consistency. Did the medical librarian on the team provide insight? I keep going back to comments earlier in this review in that I would have thought truly complex search queries would be more revealing.

**We revised the text somewhat but we’re not sure how we can be clearer. The queries were designed to be more simple than complex and to include basic field searches. We focused on straightforward searches in order to be able to track any problems we might find, and this is something we would not be able to do if were to add many terms and fields to our queries. For example, if I do a title name search in ProQuest/MEDLINE and one in EBSCOHost/MEDLINE, then any differences in search results can be explained by how these vendors index title names. But if I append to that query several other fields, then I no longer have a very good way to understand the differences between the two.**
- The focus on quantity of search results / retrieval numbers over any substantive discussion of quality of those results ("what exactly did you find when the numbers didn't agree across platforms? why did those extra references / citations appear in the retrieval sets?") is a major deficiency in the paper (which you acknowledge in your limitations section).
- In an ideal manuscript, it would be advisable to have a balance of both quantitative and qualitative aspects of your query analysis; perhaps your research team could have waited to publish until that part of your work was completed as you mention this paper is meant to be the start of a larger evaluation process.
**We disagree. Additional research will be built up on this, but we think this stands on its own and will function as a building block for additional, qualitative research projects.**

- What do I tell my students and other expert searchers/ users who might read this paper? I hope you are not suggesting we search multiple Medline platforms to compare results ourselves for simple search queries, or to look for additional papers that the individual platforms were unable to find.
**We think that you tell them that MEDLINE is not MEDLINE is not MEDLINE. That the differences between platforms is multidimensional and, like you stated earlier, probably different from one year to the next as these databases are updated and as the technology changes and evolves. We think that you tell them that that if they search EBSCOhost/MEDLINE, then they should not expect the same results as someone who searches ProQuest/MEDLINE, and so forth, and that this might raise problems. We also think that you tell them that if they publish or work with others, they should clearly document which platform that they use. We added some text to help clarify this.**

- - Finally, as a medical librarian and information retrieval expert, what does this paper tell me and how does it inform my work? In the final analysis, it is not clear to me what your message is - other than to watch for variable results in simple searches on the various platforms. Perhaps you should send your manuscript to all the vendors for comment and so they can fix the problems.

**Hopefully they read this. It’s not just vendors that need to know this. Users need to know, too. And it’s also very much a holistic issue, such that the problem is not just with vendors, but with trends in scholarly publication altoghether. So others need to be aware of this, too. Ovid did reach out to us after the JMLA paper was published and offered us free access for one year to their version of PubMed/MEDLINE. We declare this in the conflict of interests, and they had no input in the study. But it shows that they are interested in this kind of literature.**
- Best of luck.

**Thanks!**

7. PLOS authors have the option to publish the peer review history of their article ([what does this mean?](https://nam04.safelinks.protection.outlook.com/?url=https%3A%2F%2Fjournals.plos.org%2Fplosone%2Fs%2Feditorial-and-peer-review-process%23loc-peer-review-history&data=02|01|sean.burns@uky.edu|a80f1f9dac874337351f08d86e97296c|2b30530b69b64457b818481cb53d42ae|0|0|637380942806042396&sdata=Vt6Lt3VzguPLMWiI%2B2kBpRMqFmQYXsPAnu8nGCebhbA%3D&reserved=0)). If published, this will include your full peer review and any attached files.


If you choose “no”, your identity will remain anonymous but your review may still be made public.


**Do you want your identity to be public for this peer review?** For information about this choice, including consent withdrawal, please see our [Privacy Policy](https://nam04.safelinks.protection.outlook.com/?url=https%3A%2F%2Fwww.plos.org%2Fprivacy-policy&data=02|01|sean.burns@uky.edu|a80f1f9dac874337351f08d86e97296c|2b30530b69b64457b818481cb53d42ae|0|0|637380942806042396&sdata=CT9nOEP08Q9BeXlnNq8Qk%2FxXYj7xHbREtgtABe2PFNU%3D&reserved=0).

Reviewer #1: No

Reviewer #3: **Yes:** Dean Giustini

[NOTE: If reviewer comments were submitted as an attachment file, they will be attached to this email and accessible via the submission site. Please log into your account, locate the manuscript record, and check for the action link "View Attachments". If this link does not appear, there are no attachment files.]

While revising your submission, please upload your figure files to the Preflight Analysis and Conversion Engine (PACE) digital diagnostic tool, [https://pacev2.apexcovantage.com/](https://nam04.safelinks.protection.outlook.com/?url=https%3A%2F%2Fpacev2.apexcovantage.com%2F&data=02|01|sean.burns@uky.edu|a80f1f9dac874337351f08d86e97296c|2b30530b69b64457b818481cb53d42ae|0|0|637380942806052389&sdata=5bR4GnBvOyCdYU%2BMBe3BwzLOsrjgNtlIo7lDFwcf9ss%3D&reserved=0). PACE helps ensure that figures meet PLOS requirements. To use PACE, you must first register as a user. Registration is free. Then, login and navigate to the UPLOAD tab, where you will find detailed instructions on how to use the tooThis is an important and major assignment. Please submit soon. The second Wikipedia assignment builds off this one.l. If you encounter any issues or have any questions when using PACE, please email PLOS at [figures@plos.org](mailto:figures@plos.org). Please note that Supporting Information files do not need this step.

*In compliance with data protection regulations, you may request that we remove your personal registration details at any time.* [*(Remove my information/details)*](https://nam04.safelinks.protection.outlook.com/?url=https%3A%2F%2Fwww.editorialmanager.com%2Fpone%2Flogin.asp%3Fa%3Dr&data=02|01|sean.burns@uky.edu|a80f1f9dac874337351f08d86e97296c|2b30530b69b64457b818481cb53d42ae|0|0|637380942806052389&sdata=gZ1S0HTkGDqKFWeub%2F6BznE7U4VcpUMzfNp7kRWZ5Os%3D&reserved=0)*. Please contact the publication office if you have any questions.*
