# Supplementary material for: MEDLINE search retrieval issues: A longitudinal query analysis of five vendor platforms
Source: PLoS One. 2021 May 6;16(5):e0234221. doi: 10.1371/journal.pone.0234221 (PMC8101950; doi:10.1371/journal.pone.0234221)
Supplement: Attachment — Submitted filename: Response-to-Reviewers.docx [file pone.0234221.s004.docx]

Reviewer #1: PLOS1 review
Please note that line numbers refer to the version that includes markup.

This paper is much better but there are still issues:

* the terms “database”, “index”, and “platform” are conflated. This makes it very hard for a non-expert (someone who does not perform medical searching as an expert) to follow the argument. Examples of conflations that must be fixed are below. It would help tremendously if you started out by defining these three terms and then using them consistently and rigorously throughout.

We have made some effort to clarify the differences in the introduction by describing how these systems function. We think this is clear. We also would like to push back a bit. PLOS publishes thousands of articles each year in a number of different subject areas. Many of these published articles discuss specialized areas of research. While we think it’s important to write clearly, and we thank you for helping us clarify some of our language, we are not writing for the general public, just as the thousands of articles that are published PLOS are not written for the general public. We think that health science librarians and informationists and others in the broader bioscience fields who uses these systems will understand this article.

* As one of your reviewers pointed out, library experts already know all this – in detail. I keep suggesting that your paper be revised rather than rejected because I believe it is important that non-experts also understand your findings. You are still not writing for that non-expert audience.

We think this is an editorial position and that it does not affect the soundness of this research. Therefore, respectfully, we think this opinion is out of bounds.

Our goal is to write and communicate this research for the “library expert” audience, but we are also writing for the medical, bioscience, and like audiences, too. These audiences should already be familiar with MeSH, PubMed, MEDLINE, etc., as it is a regular part of medical training. Beyond that training, medical doctors and like also conduct systematic reviews, and are familiar with MeSH, etc already. And so we strongly contest the argument that this should be written for a non-expert audience or that the non-expert audience is limited to who you may be imagining. And with all due respect, we reiterate that we do not think it is fair that this paper is being judged this way. There are thousands of papers on PLOS ONE that are highly technical and scientific. We’re not sure, therefore, what is motivating this need to appeal to a non-expert audience or why you think that the expert audience is rather limited. Also, even though one of the reviewers pointed out that “library experts already know all this”, we have also shown, and rebutted in our last review, that they only know this anecdotally. Anecdotal knowledge is not scientific knowledge. This should be better evidence than anecdotal. And even if it is known among “library experts”, many of these experts still are not clear in their papers which version of MEDLINE that they are using, as we have discussed in the paper, which is why we make it clear that they should do that in their papers when we address this in our discussion and conclusion. This misunderstanding or lack of knowledge about the problems with MEDLINE also are not necessarily known among other users of MEDLINE, etc., who are experts in some ways with it or do know the basics, but who may not know the problems that we are addressing here.

My suggestion is that you write a paragraph in the introduction to make the following delineations:

Medline bibliographic records
-------- comprise -------
the Medline database
------- which is -------
indexed in different ways
------- by ----------
platforms
------- used for search and access for Medline records -------------

Thank you for your suggestion, but we believe it is clear as it stands.

The point is that access and search over what should be a stable set of Medline records is unreliable and inconsistent depending on anecdotal factors your study has quantified. It might help to think about these issues as if you were a computer programmer who is familiar with the concept of database records, indexing, and information retrieval over structured records. While there are nuances in the way librarians think about and talk about these distinctions, it is often very hard to follow a discussion that conflates these things.

We believe using computer programmer language would only confuse the issue. It’s already difficult to use terms like “database”, which has multiple meanings. We have made every attempt to be clear in our language and we believe we should not be faulted for how people with different field backgrounds might read this.

* You introduce “MESH” on line 64 but do not define or explain it. Drop the reference to LCSH – it is unimportant to your point. Non-expert readers will not make the connection between these two things. Use the space to explain what MESH is and why it matters. Below I also point out places where understanding the MESH concept is critical.
We have dropped the reference to LCSH in this sentence and have added an extra clause that further define MeSH.

* Similarly, in the next two paragraphs you refer to ERIC, DOE, NLM, etc.. Stick to the point you are making without drawing parallels – for your non-expert reader these won’t be meaningful. They just get in the way of understanding your point. Your reader needs to understand the major parts (records, database, index, platform) in order to understand how the system breaks down on a longitudinal basis. By the end of the intro these components should be clear to someone who has never heard of Medline.

We have dropped the references to these parallel systems.

* line 75: don’t say “presumably” – your reader is counting on you to say something does, may, or does not pertain; also, “access points” is librarian talk --- these are index fields

We used the term “access” only and have removed points.

* line 132: don’t say “It seems that” – same point, your reader is counting on you to say something does, may, or does not pertain. Is it true that “many health science informational professionals and librarians are aware of the
133 issues that we report here”? – then just say that.
Revised.

* Much of the material in paragraphs between line 67 and line 81 can be simplified and combined with the clear paragraph that runs from 82 to 89. As suggested above, the parallels are not helpful to your explanation.
Revised and combined.

* You have presented PubMed as a platform in most of the paper but in the introduction you imply that it is an intermediate step between the Medline index and the proprietary platforms. The role of PubMed is muddled. I have thought of two options – and I like my second idea best.

1) Maybe you can refer to the PubMed WEBSITE as the platform and simply indicate that MESH terms are added to the Medline index before it is distributed to vendors. The fact that the MESH-enhanced index “base” is called PubMed just adds needless confusion about details that are less important (paragraph between lines 82 and 89). This is related to my point on the figures – the nuances are confusing, don’t help your reader understand, and detract from the important findings.
   OR
   2) Justify using the MESH indexed PubMed as the standard against which other platforms are measured. I think this is a better approach. In this case you need to explain that the MESH-enhanced PubMed index is the foundation used by the other platforms. This makes sense, but you need to get into some version of this:

   Medline bibliographic records
   -------- comprise -------
   the Medline database
   ------- which is indexed using MESH terms to create
   the PubMed database
   ------- which is further enhanced with additional index fields by the ----------
   platforms (including PubMed WEBSITE)
   ------- that make Medline records accessible for searching -------------

   In any event, you are referring to PubMed using two difference constructs and it is confusing. Rather then try to explain the confusion, just use language that clearly separate the two rolls of “PubMed”.

We think our discussion of PubMed is clear as it stands.

* These sentences are very unclear (line 103 – 106): “If searchers used too few databases to conduct literature searches [32–34], then they may miss relevant studies [10,35]. … This is especially important in cases where data from past research is collected, analyzed, and synthesized based on published and/or gray literature,…” – Is this referring to too few platforms? Or are you saying that searchers must use Medline, additional databases, gray literature? These passages are not helping your reader. They go off the track of thinking about how variability in indexes and platforms affects query performance regarding longitudinal consistency. The point of this paragraph is very muddled.

We have divided this into two separate paragraphs and have revised to make it clearer.

* At line 134 you state “We are not aware of what the broader bio-science and medical communities know because few studies have included queries that were designed to test reproducibility be reproducible across systems.” The sentence does not make sense? You’re not aware because of the lack of studies? The revised paragraph is muddled. One issue is that non-experts have little or no information. The other is that prior studies have not been done to measure and confirm the anecdotal knowledge/reports. Both points are important and both are lost in the first part of the paragraph.

We inserted the anecdotal sentence here based on the other reviewer’s comments in the prior review and that you reference above, but this is also anecdotal – that is, whether such library experts are even anecdotally aware of these issues, or if many are, how many or how few are. In many ways, these peer reviews have been very helpful, but they are also causing some of what we are saying to get muddled. So we have removed the anecdotal sentence here because we think it’s caused some of the confusion that you point out here. Besides, we are not sure how well this is anecdotally known, even though the other reviewer claims that it is well known.

* You must explain the concepts of “MESH branch” and “exploded MESH” in language that a database designer, information science person, or computer scientist would use. Understanding your method and results depends on understanding of these concepts. Relate the ideas to the more general notions of index terms, tags, and tree structure.

* The methods section is much easier to follow. Tweaks:

** line 182 “(e.g., all queries including a single keyword search in a journal title field in a case)” is confusing and not needed

Removed.

** line 186 “were designed to search all five MEDLINE platforms” - to search EACH of the MEDLINE platforms

Revised.

** line 189 – that the data WERE collected

Revised.

** lines 193 – 202 – my version: We designed our 29 cases using basic search fields to examine compare search retrieval counts on each of the five MEDLINE platforms, therefore, our queries were designed to be short, logically clear, and to enhance the comparability of the results. For example, Table 1 describes a simple MeSH search, limited by publication date range.

Revised.

** in the lines that follow you report your results as if this is a justification for the method. You can state this more convincingly with something like this:
Our prior findings [cite yourself if this is true – our cite someone else – or simply say “based on our preliminary testing”] indicated that a wide number of records would be retrieved by different platforms, particularly when MESH terms were added to the strategies/queries. On this basis, we created 29 cases then to test a broad range of basic search strategies.

Revised.

** line 235 – “We used the modified z-score to locate deviations around results from the PubMed MEDLINE platform” – this must be justified. Why is PubMed the logical standard? (see above). Explaining this will go a long way toward making the results easier to understand. This is about the indexes. Somewhere be sure you are clearer that indexing and query structure go hand in glove. This helps explain index differences as a source of critical variability in results. Upload timing and errors are easy to understand. Indexing and retrieval differences are not.

Revised.

Conflations of “database”, “index” and “platform”:
* In your abstract, 22-23 “Specific bibliographic databases, like PubMed and MEDLINE….” – but you’ve set this up with MEDLINE as the database – and you present PubMed as one of several platforms. Conflating them in the abstract is very confusing.

We do not think we are not conflating these terms. In the first sentence, this is clear with “… of the MEDLINE database hosted on multiple platforms”.

* line 31 “Bibliographic databases are used to identify and collect research data… ” – here you are talking about platforms, not databases

“Bibliographic databases” is a legitimate term for this. Here we are not referring to any platform but to bibliographic databases generally.

* line 33 “These systems, in particular, PubMed and MEDLINE, are used to inform clinical….” – just say “these systems” to avoid confusion about the differences between PM and ML. Stick with the simple, clear and correct but incomplete definition of Medline as a core underlying database of bibliographic records and PubMed as a platform that has its own index of the Medline records

Removed “These systems”, but PubMed is more than MEDLINE and includes records not indexed in MEDLINE.

* line 36 “…the development of bibliographic databases or…” – the development of bibliographic platforms

This is not necessary to change. Platforms are much more than the bibliographic databases. Here we are referring to databases.

* line 58 “…bibliographic databases, such as those available on…” – you are talking about indexes here

We are talking about databases that exist on the platforms. We lightly revised.

* lines 62-63 “In some specialized databases, these records may be supported” – you are talking about indexes here
No, a database creates an index. We’re talking about databases. Our language is correct.

* line 90 “This differentiation among database platforms has” – this is differentiation between platforms
Revised.

* line 97 “the same MEDLINE data file” – same MEDLINE database
Revised.

* line 101 “Although the choice of database systems impacts potential source” – this is the choice of platforms
Revised.

* line 102 “it is not known how searching the sdame database (e.g., MEDLINE) on different platforms” – simplify this === “it is not known how searching .. the MEDLINE database… on different platforms … ” – that’s what you are talking about

Revised.

* line 117 “principles are applied by database vendors in indexing” -- these are platform vendors – or just platforms

Revised.

* line 120 “problematic in bibliographic databases purchased by libraries” – bibliographic platforms – ore more generally “bibliographic resources” – reserve “database” to refer to Medline only
Revised.

* line 125 “license these records to database vendors to host on their own platforms” – to vendors – you don’t need to call them database vendors
Revised.

Finally, your results section is still extremely unclear and profusion of charts is distracting and confusing. All this seriously undermines your point. The notion of macro and micro are also not useful. Rather then giving these two perspectives unnecessary and confusing names, just write what what you are talking about – what you did. The idea of analysis “scale” is also not useful. In order to understand what you are reporting I need to revert to looking at your raw data. In short, major changes are still needed to make the results meaningful for non-expert readers. I believe only a determined expert will be motivated to decipher it.

We disagree that this is confusing and that the terms we’re using are not useful. We already know that our results have been looked at based on the comments via social media about our preprint (<https://www.biorxiv.org/content/10.1101/2020.05.22.110403v1>) and that our points have been understood. Figure 3 is an important contrast to Figures 4, 5, and 6, and the terms “micro” and “macro” have no special meaning beyond their everyday definitions. However, based on the other reviewer’s comments, we have clarified the language and research involving the macro and micro views.

In general, Figure 3 is meaningless. You can show one example if you wish, but the text should convey that whatever it is you are measuring in those charts is a constant month over month. The results displayed in Figures 4, 5 and 6 are meaningful, but the meaning is buried in the complexity of the text and in all the charts. In my opinion, your results section should focus on making very clear the meaning and implications of data displayed in figs. 4, 5, 6, but you need to select only one chart as an example for each type of anomaly you have found. You can make all the other charts available online with the data. The details in figures 7 and 8 are also obscure your point. You can report that information much more effectively with one example figure and a summary table on your stats.

We strongly disagree that these figures are meaningless or that they obscure our point. If you feel you need to recommend rejecting this article for these reasons, I guess go ahead. We’ve already defended these plots and we think they are important visualizations.

Many of the details of your results section are dense and hard to understand, particularly without definitions of things like MESH and “explode”. If there are important esoteric nuances to the specific cases, that detail may be better suited for publication in a second paper written specifically for experts.

Again, we think this criticism is unfair and does not impact the soundness of our research. We are not writing for the general public, just as most articles on PLOS are not for the general public. We appreciate your recommendations above and previously. You have helped us clarify the language and the terms, which are confusing for a number of reasons beyond this research. But we can only go so far in making this paper accessible to the “non-expert”.

With a greatly simplified presentation of your results readers will be able to follow. This will make your final chart (Fig 9) much more meaningful. This is a nice, clear and current demonstration of the effect of the issues you’ve characterized.

Reviewer #3: This is my second review of your paper. You and your team have made progress in some areas of the paper; some parts of the paper are coming together, but other parts are still unclear.

I've made extensive editorial comments (N=34 in total) focusing on using simpler, more consistent language in the manuscript. Some sections in the paper are still difficult to understand; I've done my best to try to assist in clarifying your points but not certain I've achieved much. Best of luck with your research.

My comments are:

1) I like the new title for the article, "MEDLINE search retrieval issues: A longitudinal query analysis across select vendor platforms" though I would take out the word 'select' and replace it with the word, "five".

Revised.

2) Abstract: Your first sentence is too long and somewhat confusing. Can you consider a change to the second sentence at line 14 as, "We devised twenty-nine search queries, or cases, comprised of five semantically equivalent queries per case to search against five MEDLINE platforms." Lines 17-23 are important and I'd recommend breaking up the section by saying, "We found that search results varied by MEDLINE platform within sets and across time. Reasons for variations were due to trends in scholarly publishing such as publishing individual papers online first versus complete issues in print format. Some other reasons were metadata differences in bibliographic records; differences in levels of specificity of search fields across platforms and large fluctuations in monthly search results based on the same query. Database integrity and currency issues were observed as each platform updated its MEDLINE files throughout the year."

Revised.

3) Line 35 should say "...in the health professions and to construct a knowledge base for clinical decision support systems".

Revised.

4) Line 49 remove "should".

Revised.

5) Line 53 sounds a bit odd with "relies"; I would use rely for better agreement.

Revised.

6) Lines 54-58 could be shortened as "The Preferred Reporting Items for Systematic Reviews and Meta-Analyses (PRISMA) Guidelines and the Cochrane Handbook for Systematic Reviews of Interventions provide examples for scholars in their reporting of methods and organizing of reviews".

7) At line 60, it's not that they rely on structured records but that they are designed that way for descriptive purposes and to create reliable post-hoc searchable citations/ records.

Revised.

8) This sentence is unnecessarily awkward at line 61, "These bibliographic records contain fields we take as meaningful for providing discovery and access, such as author name fields, document title fields, publication title fields, and date of publication fields." Perhaps take out "we take as meaningful'.

Revised.

9) At line 65, I would not use this phrase, "are based on standard knowledge classification efforts". I would not refer to subject analysis and/or article indexing as knowledge classification for obvious reasons.

Revised.

10) Line 75, I don't really understand why you use the phrase "Commercial information service companies" when you can say simply "Commercial vendors such as" or simply "vendors". I wouldn't end the sentence at line 78 with a preposition. I would say "database providers, such as the NLM or DOE, etc" and remove the word 'original' which is not accurate in any case.

Revised based on your comment and the other reviewer’s.

11) Line 78, isn't it really about database "features" and not "value"? What do you mean by value? Not sure I agree that it's about value and not about searchable features.

Just that adding features that are different than what can be found on PubMed is a selling point for them. But revised.

12) Commercial information service provider at line 86, say "vendor". You don't need to keep changing the phrase which will only confuse your reader. (You also continue this practice throughout the manuscript).

Revised throughout.

13) Line 90 why say "differentiation"? when you can say "Differences in features across platforms"? And I'm not sure that accounting for those differences is why we report the specific platforms, interfaces and / or vendors. It's to help reproduce the searches as reported by using the correct platform.

Revised.

14) Line 97 I would remove "presumably". It's a kind of cynical word to use in this context.

Revised.

15) Line 101: this sentence seems to relate more to the previous sentence than what comes after it.
Revised this section.

16) Line 114: "database systems" should be vendor or "vendor systems" or "database vendors". If clarity is needed, you can say "Vendor systems / or vendors such as OVID or EBSCOHost".

Revised.

17) Line 121: I'd remove "also". There are too many examples of unneeded uses of this word in your paper; many are not needed.

Revised.

18) Line 132: Remove "It seems that" and the sentence needs a citation. I would argue that varying results by vendor platform have been seen in other papers and you could cite one or two. Cite your previous paper from JMLA for example.

Revised based on this and other other reviewer’s comments.

19) Line 144: Change this sentence to something along the lines of "This paper builds on that work and examines differences over time by evaluating longitudinal data, which is a critical factor in replicating bibliographic database search results. "

Revised.

20) Line 162: This first sentence is terribly confusing to me. I would say, "We designed 29 cases, or sets, of searches which comprised five (5) semantically similar or equivalent queries to perform searches in five MEDLINE platforms for a total of 145 searches".

We have taken out the term sets because another reviewer didn’t like that term and suggested using cases. Otherwise revised.

21) Lines 180-188: can you say here why you picked dementia? Because it's one word? Because it is short and easy? Some rationale here is really needed even if you feel you are repeating information. It will help your reader understand why you picked it and grouped it with neoplasia.

Revised. Chosen just based on simplicity and for representation. The term was not grouped with neoplasms. Those were separate searches.

22) Line 247: Can you set up this section for clarity? For example, "The data gathered in the majority of our searches (20 cases of five searches each for a total of 100 queries) did not include limits on publication dates. However, we did include date delimited searches for nine cases of five searches for a total of 45 queries. We used publication date limits from 1950 to 2015 for these search queries. We found that...etc."

Revised.

23) Line 259: This sentence is awkward. Can you say simply, "Second, based on a detailed examination of the same cases...". Remove the use of the word also (2x) from lines 263 and 268.

Revised.

24) Line 272: This whole section on macro/micro is more confusing than it needs to be. I would consider using more simple language. For example "We examined the data of the cases, and present variations we identified based on two categories which we will use in the following section: an overview (macro) of the data and a more granular (or, micro) view of the variations arising in the data".

Thank you. Revised.

25) Line 323: I would relabel this section as "Online first and print publications impact bibliographic control". Also line 327: I would avoid the phrase "non exploding". Can you say "...not exploded, but differed because case #04 is restricted by publication date". Please use simpler sentence construction for the benefit of communicating your results. Throughout this section, I would look critically at using the phrase "non exploding" as this is not the way this is referred to in describing the feature. Say "not exploded".

Revised throughout.

26) Line 339: I would not say "indicates"; perhaps "suggests" would make more sense.

Revised.

27) Line 348: Change along these lines, "The record indicates it was added to PubMed in 2015 but not formally entered into MEDLINE until 2019".
Revised.

28) Lines 361-378 should be edited for brevity. It's also very acronym heavy.
Revised.

29) Line 379: what do you mean by the title of this section "Reproducible with specific field searches"? Do you mean "Reproducibility improved with search specificity (or field searching)"

Yes. Revised.

30) Lines 394-397: the label for this section is a bit unclear as is the first sentence of the section. Perhaps it's the use of the word "outliers". What does this mean? Just different from the rest? An error? An incorrectly inflated number?

Revised.

31) Lines 400-522: this section is quite difficult to read. Without going into detail, I'd recommend a close edit. For example, at Line 488-490, try something simpler such as "We could not identify the reasons for the significant variance in search results over time. As such, searchers may be left wondering why they are seeing variances in search results which may further undermine their trust in using the different platforms".

Revised.

32) In your Limitations section, you need an introductory sentence. "Our paper has a number of limitations based on X, Y and Z." As it's written in the first sentence, I don't understand what you mean by saying that using PubMed / Medline is a limitation (due to different coverage and content or...?).

We removed the first paragraph.

33) Line 523: why is documenting variances in systematic searches across platforms a useful area of research? Could you explain this to the reader? It might be important - but your explanation doesn't really clarify. Perhaps you could say that documenting variances on platforms is NOT as important as trying to identify which vendor platform is the most reliable over time, and why. That's the issue we as searchers want to know about and why we are reading your paper. Perhaps a ranked list would be a good research project. It shouldn't be up to health librarians or expert searchers to document these variances. They take up our time! to do searches. I think the semantically selected search queries are a limitation also but you don't really know because you haven't tested them. You might say this may have affected generalizability.

Revised and explained. We think we explained the importance in the beginning of the paper but we have attempted to tie up that loose end. Regarding the semantic issue, we’re not sure what you mean. But also, this is not something that will be generalizable, as we discussed in the previous review. The systems need to be standardized.

34) Conclusions in your paper: Not sure why you introduce Google Scholar in your lead sentence of your conclusion. I'd reconsider that first sentence very closely. Also, what's the most important concluding remark about your research after all the work you put into it? Perhaps it's that "No single MEDLINE platform emerged as the most reliable interface to search for citations for this project". The data suggests that there were greater variances in search results over time for simpler, shorter search queries and that the more sophisticated search queries performed more reliably over the five vendor platforms. Whatever it is, can you be explicit? Best of luck.
/dg

We say that this study indicates that there is no one MEDLINE. We’re not sure the critique about the analogy to Google Scholar.
