# Supplementary material for: MEDLINE search retrieval issues: A longitudinal query analysis of five vendor platforms
Source: PLoS One. 2021 May 6;16(5):e0234221. doi: 10.1371/journal.pone.0234221 (PMC8101950; doi:10.1371/journal.pone.0234221)
Supplement: Attachment — Submitted filename: Response-to-Reviewers.docx [file pone.0234221.s005.docx]

Dear Editor,

We have accepted all the suggestions from Reviewer #3 below, and per their additional advice, have thoroughly edited the manuscript. We would like to extend our gratitude to you and the reviewers for their close inspection of the manuscript and for their patience with this review process.

Sincerely,

C. Sean Burns

Reviewer #3: Dear Authors,
This is now my third review of your manuscript, now entitled "MEDLINE search retrieval issues: A longitudinal query analysis of five vendor platforms". I agree your paper is improved but wonder if it would benefit even further from another vigorous editing. Show it to a copyeditor because it's important research. (It's possible the peer review process has forced you to make changes to your paper that conflict in some way with what you are communicating, which has happened to me before.)

In any case, here are some of my suggestions for your consideration:
1. Abstract, first sentence: Could you not say, "This study compares results of a longitudinal search query analysis of
MEDLINE hosted on five (5) platforms: PubMed, EBSCOHost, Ovid, ProQuest, and Web of Science." In reading the sentence as it is currently, it sounds as though you are covering more than five platforms. ("....that include").
2. Abstract, fifth sentence: could you put in past tense? "We found that search results varied considerably depending on MEDLINE platform."
3. Abstract, later sentence: Could you not say "biomedical" instead of "bioscience"? Otherwise you have to define what you mean by this unusual word. Do you mean biomedical sciences? Instead, how about: "....Specific biomedical bibliographic databases are used to inform clinical decision-making, create systematic review searches....etc."
4. Abstract, last sentence: could you not simplify as "They serve as essential information retrieval and discovery tools to help identify and collect research data, and are used in a broad range of fields as the basis of research design. This study should help clinicians, researcher, librarians, informationists, and others understand how these five platforms differ and inform future work in their standardization." (I have not heard "informationalists" before and didn't catch this in the earlier version.)
5. Introduction, first paragraph, this is your all-important entry point for your reader. Could you consider something along the lines of, "Bibliographic databases are used to identify and collect research papers and function as a critical part of scientific investigation. Studies that employ bibliographic databases include research on bibliometrics/scientometrics, information literacy, systematic reviews, and meta-analyses, to name a few. In particular, PubMed and MEDLINE are used to inform clinical decision-making by health professionals and in building knowledge bases for clinical decision support systems". (I see you cite the Hines (2006) paper but remember that study is about genomic databases not bibliographic databases; I would not extrapolate and say that bibliographic databases are scientific instruments. Sorry I didn't capture that beforehand.)
6. Line 41, remove the extra "to"s...."Researchers, librarians, information scientists, and others rely on bibliographic databases to conduct research, instruct future information and other professionals on how to conduct literature searches, and assist those with information needs to locate and access literature"
7. Line 44, this sentence will confuse your reader and should be simplified. Could you consider, "Furthermore, these databases have standard rules to describe research papers, and are structured using controlled vocabularies, in order to make searching for information more precise or comprehensive".
8. Line 47, could you say "Fine control over bibliographic searching and documentation of search strategies, which are reported in systematic reviews, allow for the replication and reproduction of searches."
9. Line 61, could you break into two sentences such as, "In certain specialized databases, these records are often supported by a set of thesaurus terms, or a controlled vocabulary, such as the Medical Subject Headings (MeSH) in the MEDLINE database. Their goal is to describe the subject matter of works or records that are indexed in the bibliographic database to assist in consistent information retrieval."
10. Line 67, amend slightly as "The MeSH thesaurus is freely available on the U.S. National Library of Medicine's (NLM) PubMed website, and is used to search MEDLINE on other platforms such as EBSCOhost, Ovid, ProQuest, and Web of Science."
11. Line 70, I would say MEDLINE here not PubMed. Vendors buy MEDLINE not PubMed, "These commercial vendors provide access to the bibliographic data from MEDLINE and the corresponding MeSH thesaurus and add features on their respective platforms beyond what NLM has already provided."
12. Line 72, repetition of "their" can be removed and amended, "The added features are based on a vendor's unique user interface, search features, ability to link to library collections via proxies, related additional database content, or searching multiple databases on a specific platform in a single search session."
13. Line 76, paragraph amended along the lines of, "However, these added features may create some differences in searching and in the search results [26,27]. For example, MEDLINE can be searched using PubMed which is defined by the nearly 6000 publications it indexes, structure of its bibliographic records, use of MeSH in those records, and its freely-available search interface on the web. When vendors provide access to MEDLINE, they start with the MEDLINE system but creating a customized subscription-based version that includes a new interface, slightly different search fields, search operators, and other features."
